# Selective intra-arterial infusion of rAd-p53 with chemotherapy for advanced oral cancer: a randomized clinical trial

**DOI:** 10.1186/1741-7015-12-16

**Published:** 2014-01-30

**Authors:** Yi Li, Long-Jiang Li, Li-Juan Wang, Zhuang Zhang, Ning Gao, Chen-Yuan Liang, Yuan-Ding Huang, Bo Han

**Affiliations:** 1 State Key Laboratory of Oral Disease, West China Hospital of Stomatology, Sichuan University, Chengdu, PR China; 2 Department of Head and Neck Oncology, West China Hospital of Stomatology, Sichuan University, Chengdu, PR China; 3 West China Health Hospital, Sichuan University, Chengdu, PR China

**Keywords:** Oral carcinoma, Gene therapy, Chemotherapy, Intra-arterial infusion, p53

## Abstract

**Background:**

In this study, a combination of recombinant adenoviral p53 (rAd-p53) gene therapy and intra-arterial delivery of chemotherapeutic agents for treatment of oral squamous cell carcinoma was evaluated.

**Methods:**

In total, 99 patients with stage III or IV oral carcinoma who had refused or were ineligible for surgery were enrolled in a randomized, placebo-controlled, double-blind, phase III clinical trial. They were randomly assigned to group I (n = 35; intra-arterial infusion of rAd-p53 plus chemotherapy), group II (n = 33; intra-arterial infusion of rAd-p53 plus placebo chemotherapy), or group III (n = 31; intra-arterial infusion of placebo rAd-p53 plus chemotherapy).

**Results:**

The median length of follow-up was 36 months (range, 3 to 86 months). During follow-up, 16 patients in group I, 20 in group II, and 22 in group III died. Group I (48.5%) had a higher complete response rate than groups II (16.7%) and III (17.2%) (*P* = 0.006). The rate of non-responders in group I was significantly lower than that in groups II and III (*P* < 0.020). A log-rank test for survival rate indicated that group I had a significantly higher survival rate than group III (*P* = 0.019). The survival rate of patients with stage III but not stage IV oral cancer was significantly higher in group I than in group III (*P* = 0.015, *P* = 0.200, respectively). The survival rate of patients with stage IV did not differ significantly among the three groups. Or the 99 patients, 63 patients experienced adverse events of either transient flu-like symptoms or bone marrow suppression, while 13 patients had both these conditions together. No replication-deficient virus was detected in patient serum, urine, or sputum. rAd-p53 treatment increased Bax expression in the primary tumor of 80% of patients, as shown by immunohistochemical staining.

**Conclusions:**

Intra-arterial infusion of combined rAd-p53 and chemotherapy significantly increased the survival rate of patients with stage III but not stage IV oral cancer, compared with intra-arterial chemotherapy. Intra-arterial infusion of combined rAd-p53 and chemotherapy may represent a promising alternative treatment for oral squamous cell carcinoma.

**Trial registration:**

ChiCTR-TRC-09000392 (Date of registration: 2009-05-18).

## Background

Oral cancer is a major global health problem with 300,000 new cases diagnosed each year. Alcohol and tobacco remain prominent etiologic factors in oral cancer [1], but high-risk human papillomavirus (HPV) has emerged as an important etiologic agent [2]. Several potentially curative surgical and non-surgical treatment options exist for patients with oropharyngeal cancer [3,4], but treatment selection (chemoradiotherapy and primary surgery) is complex, reflecting the need for primary tumor and nodal disease control to be balanced against preservation of function and minimizing long-term toxicities. Despite advances in treatment with surgery, radiotherapy, and chemotherapy, this aggressive epithelial malignancy is associated with severe morbidity, and patients have a long-term survival rate of less than 50% [5,6]. Treatment failures mainly involve development of second primary tumors in patients with early stage disease (stages I and II), and local recurrence and metastases in patients with locally advanced disease [7,8]. New approaches are urgently needed to improve the outcomes of patients with oral cancer.

Gene therapy with wild-type human tumor suppressor p53 (wt-p53) is a promising treatment strategy for some malignant tumors [9-12]. The wt-p53 protein is a primary mediator of cell cycle arrest, DNA repair, and apoptosis. The mutation and inactivation of p53 may be a crucial event in the origin and progression of head and neck carcinoma [13-15], and potentially contributes to the development of drug resistance in tumors of epithelial origin [16]. The incidence of p53 mutation is approximately 31% in squamous cell carcinoma (SCC) [17,18]. Introduction of the wt-p53 gene inside tumor cells may restore tumor suppressor functions and improve treatment outcomes in some patients [12,15,19]. Reintroduction of wt-p53 has been accomplished with recombinant adenoviral p53 (rAd-p53) [20]. Adenoviral delivery of wt-p53 results in strong wt-p53 protein expression in tumor cells, with minimal hematopoietic toxicity [10,11,13,14,17,19-27]. Clinical trials have demonstrated that rAd-p53 is effective against a variety of malignancies [10,11,13-15,17,19-27]. Routes of administration typically used for rAd-p53 are intratumoral injection, perfusion, and intravenous infusion [15,28-33]. Although intratumoral rAd-p53 combined with radiotherapy has led to higher rates of locoregional control [34], and rates of complete response (CR) were up to 75% of patients by 12 weeks in a phase II study [32,33], rAd-p53 alone has led to CR in only a few cases [12,28]. Although intratumoral injection delivers the recombinant adenovirus to its target, the spread of the recombinant adenoviral vectors from the injection site is minimal [35].

Several studies have examined the use of selective intra-arterial infusion of chemotherapy for head and neck cancers [36-39], and suggest modestly higher responses or at least non-inferiority [39-41]. Potential benefits of intra-arterial infusion include more precise delivery to the target organ, potentially wider dissemination in the tumor than occurs with intratumoral injection, and fewer systemic side effects [39]. Intra-arterial chemotherapeutic administration can also provide high response rates and acceptable palliative treatment without surgery or radiotherapy in elderly patients [39]. Because a high concentration of rAd-p53 can be delivered to an oral tumor through intra-arterial infusion with minimal systemic toxicity, the aims of this randomized, placebo-controlled, double-blind, phase III clinical trial were to compare the efficacy and safety outcomes of intra-arterial infusion of combined rAd-p53 and chemotherapeutic drugs with those of the single treatments individually for treatment of oral carcinoma. This study also assessed the expression of p53 and two transcriptional targets, Bax and Bcl-2, in the biopsy specimens, using immunohistochemical analysis.

## Methods

### Patient selection and randomization

This randomized, placebo-controlled, double-blind, phase III pilot study of intra-arterial rAd-p53 with or without chemotherapy recruited 248 patients with advanced oral SCC between 2003 and 2007 from a single center (West China Stomatology Hospital). The study center was not a specialist referral center. Diagnosis of advanced disease (stages III and IV) was performed by three independent physicians experienced in oral and craniofacial surgery using World Health Organization (WHO) head and neck malignant tumor classification criteria, including patients’ clinical performance, clinical examination, and computed tomography/magnetic resonance imaging (CT/MRI). These stage III or IV oral cancer patients were ethnic Chinese from five provinces in southwest China, reflecting a wide geographic area, and they had refused or were not eligible for surgical treatment.

Of the patients assessed, 149 patients were excluded for one or more of the following reasons: 1) systemic disease; 2) adenocarcinoma; 3) history of other treatments; 4) financial or logistical barriers to completing the trial; and 5) refusal of informed consent. Specifically, 68 patients were excluded for severe systemic disease (32 with cardiovascular disease, 15 with chronic obstructive pulmonary disease, 16 with hematologic disorders, 3 with liver dysfunction, and 2 for other reasons); 33 patients had received previous cancer-related therapy; 12 patients refused treatment for financial reasons; 17 patients had adenocarcinomas; and 19 patients did not give informed consent.

**Figure 1:**
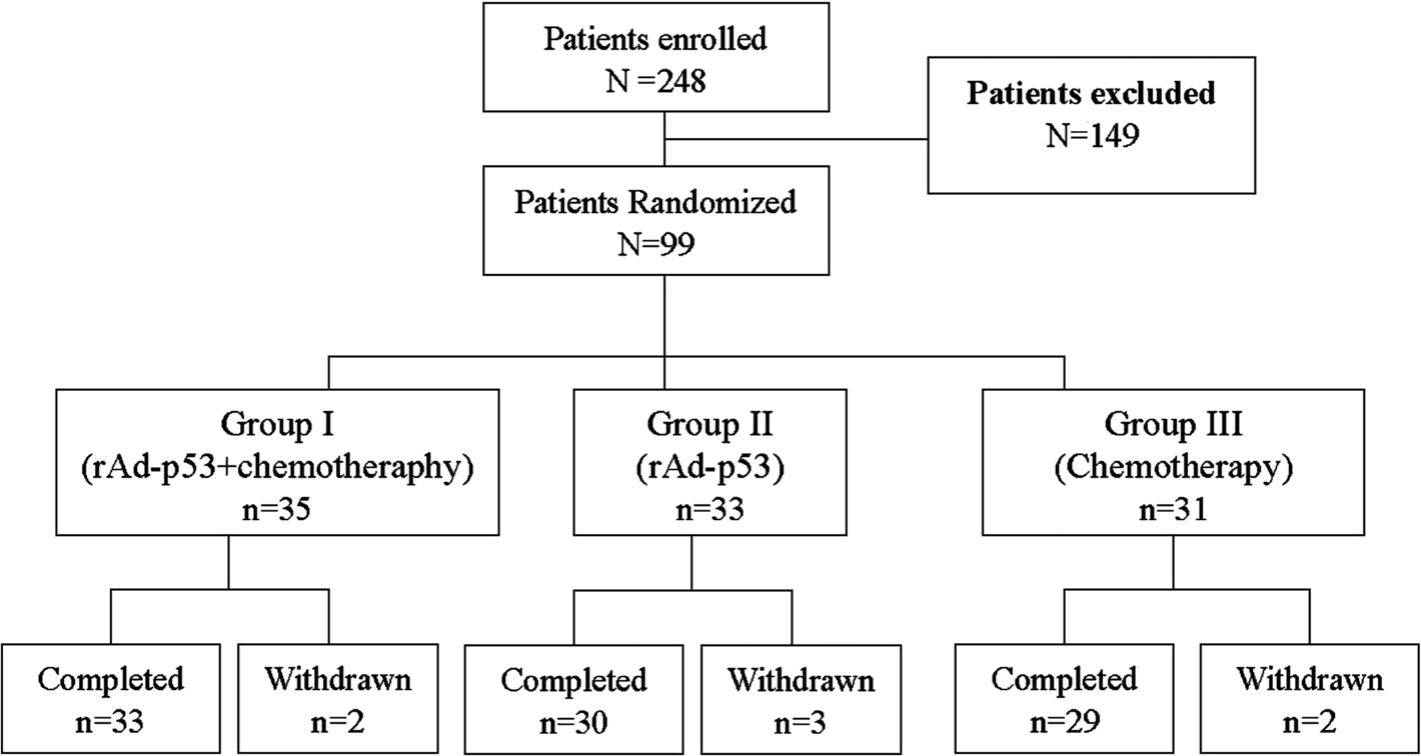
Flow diagram of the clinical trial procedure.

This left 99 treatment-naive patients with advanced SCC (29 stage III, 70 stage IV) to be enrolled in the study, and they were randomly assigned to three treatment groups: group I (n = 35; rAd-p53 plus chemotherapy); group II (n = 33; rAd-p53 plus placebo chemotherapy); and group III (n = 31; placebo rAd-p53 plus chemotherapy). Patients were randomized to the groups in a 1:1:1 ratio by using SAS 9.0 to generate a list of sequential numbers randomly from a permuted-block randomization procedure with a block size of 9 (SAS 9.0; SAS Institute, Cary, NC). Group III received the current standard chemotherapeutic drugs used as non-surgical treatment in our institution, with the modification of intra-arterial instead of intravenous infusion. A flow diagram of the study is shown in Figure 1.

No patients had undergone prior cancer treatment, and none had uncontrolled infections or immunodeficiency disease. Patients returned for periodic post-treatment physical examination, with follow-up by phone and mail, reflecting our standard practice.

### Ethics approval

All enrolled patients provided written informed consent. The study was approved by the Institutional Review Board of State Key Laboratory of Oral Disease, West China School of Stomatology, Sichuan University, and was performed in accordance with Good Clinical Practice (GCP) guidelines for clinical trials, and according to the tenets of the Declaration of Helsinki.

### Selective intra-arterial infusion of rAd-p53 and/or chemotherapeutic agents

The recombinant adenoviral p53 (Gendicine™; ShenZhen SiBiono GeneTech, Shenzhen, PR China) is a recombinant human serotype 5 adenovirus in which the E1 region is replaced by a human wild-type p53 expression cassette [42,43]. The p53 gene is driven by a Rous sarcoma virus promoter with a bovine growth hormone poly(A) tail. The recombinant adenovirus is produced in human embryonic kidney 293 cells grown in a bioreactor. Virus produced from the bioreactor is further processed and chromatographically purified.

Recombinant adenoviral p53 was stored at -70°C at a concentration of 1 × 10^12^ virus particles (vp) per 2 ml ampoule. Frozen rAd-p53 was thawed, diluted in 8 ml normal 0.9% NaCl solution (10 ml total volume) at room temperature, and 10 ml were administered over a period of 20 minutes within 1 hour after thawing and dilution. Chemotherapeutic agents for treatment of SCC were carboplatin, bleomycin, and methotrexate. Intra-arterial infusion protocol (timing, volume, and administration) was identical in all three groups for active or placebo treatment. Saline solution was used for placebo infusions in groups II (placebo chemotherapy) and III (placebo rAd-p53).

The temporal region was anesthetized locally, and a long-term catheter was advanced retrogradely into the superficial temporal artery until the tip reached the opening of the main feeder artery arising from the external carotid artery. After catheterization was confirmed by infusion of methylene blue tracer, an indwelling arterial pump was implanted subcutaneously in the temporal region. Patients with advanced primary tumors that had spread to the contralateral side received bilateral catheterization. All treatments (rAd-p53 and chemotherapeutic agents, and placebo), were administered by means of an arterial pump. The same indwelling arterial pump was used to deliver gene therapy (20 minutes) and/or chemotherapy (2 hours for carboplatin; 30 minutes for bleomycin and for methotrexate). The patients’ vital signs were monitored during administration.

Patients in groups I and II received 10 cycles of rAd-p53 infusion within 6 weeks. The selected dose of rAd-p53 was delivered over 20 minutes via 10 ml syringes once every 4 days. The rAd-p53 doses were 1 × 10^12^ or 2 × 10^12^ vp per patient for unilateral or bilateral catheters, respectively. The rAd-p53 doses remained the same throughout the study. Patients were closely monitored for 4 hours after each rAd-p53 administration. Patients in group III received placebo rAd-p53 (saline solution) using this protocol, while those in group I received two courses of combination chemotherapy identical to that given to group III, and those in group ii received placebo chemotherapy (saline solution) using the identical protocol. The first combination chemotherapy began 2 days after the first rAd-p53 infusion. The treatment periods were separated by a 7-day break. After infusion, the catheter was filled with 2000 U heparin to prevent coagulation.

At the end of the study, some patients had become potential candidates for surgery or radiotherapy. Patients who experienced post-treatment recurrence during follow-up were given additional radiotherapy for salvage. To avoid a confounding effect of surgery on the outcomes of the three groups, post-treatment outcomes were assessed prior to local management or salvage therapy. Thus, the post-treatment outcomes of this study were not affected by these subsequent interventions.

### Evaluation of therapeutic effects and toxicities

We evaluated the therapeutic responses of the primary tumor lesion by clinical examination (for lip carcinomas), or by CT or MRI (for other oral carcinomas). Once the initial scan revealed response, a follow-up scan was performed within 1 month to verify response. Treatment results were assessed as follows: 1) CR: disappearance of all tumor masses for at least 1 month; 2) partial response (PR): a decrease of 50% or more in the diameter product (largest diameter × perpendicular diameter) of the measurable tumor for at least 1 month; 3) stable disease (SD): a decrease of less than 50% or an increase of less than 25% in the diameter of the lesion; and 4) progressive disease (PD): an increase of greater than 25% in the diameter product, or the development of a new lesion. Toxicities and adverse clinical events, such as fever, chill, nausea, diarrhea, vomiting, fatigue, and myalgia, were evaluated and recorded, in accordance with the guidelines from the National Institute for the Control of Pharmaceutical and Biological Products [44].

Patients in groups I and II had laboratory, hematology, and blood chemistry testing performed before treatment and within 24 hours after every rAd-p53 administration. Patients in groups I and III underwent the above examinations on the final day of each period of chemotherapy. Urine, stool, and serum samples were collected from patients in groups I and II. Serum samples were tested for the presence of anti-rAd-p53 immunoglobulin G. A cytopathic effect assay was used to detect the amount of vector disseminated in body fluids. Serum samples were analyzed for presence of anti-rAd-p53 antibody and vector within 24 hours and at 14 days after the initial infusion of rAd-p53, and at 1 month after the final rAd-p53 infusion.

### Expression of wt-p53, Bax, and Bcl-2

Biopsy specimens were taken within 3 days prior to receipt of rAd-p53 treatment and at 3 days after treatment. The specimens were immediately fixed in 10% formalin, blocked in paraffin wax, and cut into 5-μm serial sections. Sections were examined using hematoxylin and eosin staining. Adjacent sections were evaluated for expression of wt-p53, Bax (a protein induced by p53), and Bcl-2 (a protein that is down-regulated by p53) by immunohistochemistry using specific antibodies. Immunostaining of carcinoma cells was assessed qualitatively and semi-quantitatively. Semi-quantitative analysis scored the staining into the following four classes: if less than 10% of cells were labeled, it was scored as -; 0% and less than 25% staining as +, between 25% and less than 50% staining as ++, and 50% or greater staining as +++. Immunostaining of the samples was assessed by two independent blinded observers. Variations between the two observers were determined to be less than 5%. After serial dilution of serum, the anti-p53 antibody titers were detected by ELISA assay.

### Statistical analysis

Continuous variables in the three treatment groups were presented as mean ± standard deviation (SD) and compared using analysis of variance (ANOVA). Multiple comparisons between groups were performed by using the Bonferroni procedure with a type I error adjustment. Categorical variables were presented as numbers (percentages) and analyzed by the χ^2^ test or Fisher’s exact test. Moreover, ordinal data (tumor classification, International Union Against Cancer (UICC) stage, Bcl-2 expression, and tumor grading) was determined using the Kruskal-Wallis test. The paired *t*-test was used to assess the difference in minimum inhibitory dilution (MID, a measurement of rare cells and precursor white cells) and counts of whole blood cells, lymphocytes, neutrophil and platelets before and after treatment. Kaplan-Meier curves with log-rank tests were created to assess the survival rates for the three groups. All statistical assessments were two-sided. and a *P*-value of 0.05 was set as significant. Statistical analyses were performed using SPSS 15.0 statistical software (SPSS, Chicago, IL, USA).

## Results

In this randomized, placebo-controlled, double-blind, phase III clinical trial, 99 patients with stage III or IV oral SCC were randomly assigned to group I (n = 35; rAd-p53 plus chemotherapy), group II (n = 33; rAd-p53 plus placebo chemotherapy), or group III (n = 31; placebo rAd-p53 plus chemotherapy). Baseline characteristics of the three groups were similar (Table 1). All tumors were SCC (presence of adenocarcinoma was an exclusion criterion). Most tumors were classified as T3 or T4, and were well balanced across the three groups. Each group included approximately 60% of patients with stage IVa, approximately 11% with stage IVb and 30% with stage III disease, based on the UICC TNM cancer staging system. The treatments were administered by intra-arterial infusion into the primary lesion. Because of financial reasons, seven patients dropped out of the study after randomization. Dropouts occurred during the early years of the study, reflecting the financial or coverage environment in the geographic area at that time. As a result, 33, 30, and 29 patients in groups I, II, and III, respectively, completed the study.

**Table 1:** Characteristics of 99 patients accepted for treatment by gene therapy as part of their disease management

**Case information**	**Group**^**a**^			***P*-value**
	**I (n = 35)**	**II (n = 33)**	**III (n = 31)**	
Age, years, mean ± SD^b^	57.06 ± 10.63	59.79 ± 12.31	55.29 ± 10.60	0.274
Gender, n (%)^c^				0.953
Male	21 (60.0)	21 (63.6)	19 (61.3)	
Female	14 (40.0)	12 (36.4)	12 (38.7)	
Tumor classification, n (%)^d^				0.909
T2	3 (8.6)	3 (9.1)	4 (12.9)	
T3	16 (45.7)	13 (39.4)	12 (38.7)	
T4	16 (45.7)	17 (51.5)	15 (48.4)	
UICC stage, n (%)^d^				0.0.996
III	11 (31.4)	9 (27.3)	9 (29.0)	
IVa	20 (57.1)	20 (60.6)	19 (61.3)	
IVb	4 (11.4)	4 (12.1)	3 (9.7)	
Tumor site, n (%)^e^				1.000
Tongue	13 (37.1)	11 (33.3)	12 (38.7)	
Buccal membrane	10 (28.6)	10 (30.3)	8 (25.8)	
Gingiva	6 (17.1)	7 (21.2)	7 (22.6)	
Floor of mouth	3 (8.6)	2 (6.1)	2 (6.5)	
Palate	1 (2.9)	1 (3.0)	0 (0.0)	
Lips	2 (5.7)	2 (6.1)	2 (6.5)	
Bax expression, n (%)^e^				0.796
< 1% (-)	30 (85.7)	30 (90.9)	27 (87.1)	
10 to 25% (+)	5 (14.3)	3 (9.1)	4 (12.9)	
Bcl-2 expression, n (%)^d^				0.241
<10% (-)	2 (5.7)	5 (15.2)	5 (16.1)	
10 to 24% (+)	4 (11.4)	10 (30.3)	4 (12.9)	
25 to 49% (++)	15 (42.9)	9 (27.3)	13 (41.9)	
≥50% (+++)	14 (40.0)	9 (27.3)	9 (29.0)	
Tumor grading, n (%)^d^				0.956
I	5 (14.3)	3 (9.1)	2 (6.5)	
II	14 (40.0)	15 (45.5)	15 (48.4)	
III	16 (45.7)	15 (45.5)	14 (45.2)	

^a^Group I: chemotherapy and rAD-p53; group II: rAD-p53; group III: chemotherapy.

*P*-values are based on ^b^ANOVA; ^c^χ^2^; ^d^Kruskal-Wallis test, and ^e^Fisher’s exact test.

### Treatment outcome

The primary lesions of 58 out of 92 patients responded to therapy, including 26 with CR and 32 with PD. The CR rate and the SD and PD rates were significantly different between the groups (Table 2). The response rate (CR + PR) was 27/33 (82%) for group 1, 16/30 (53%) for group II and 15/29 (54%) for group III. The PR rates were similar for all three groups, at 33 to 35%. The CR rates were highest in group I: 16/33 (48%) had CR compared to 17% in groups II and III. It is notable that 10 of 23 patients in group I with stage IV disease were categorized as having CR. The rate of non-responders (SD or PD) in group I was significantly lower than that in groups II and III (*P* < 0.020). The non-responder rate in group I patients with stage IV disease was 17.4% versus 50% in both groups II and III (*P* = 0.028). Few of the patients with stage III disease were non-responders, and the non-responder rates between the groups were not significantly different (*P* = 0.645).

**Table 2:** Clinical response of primary lesion and side effect profile for the three groups (n = 92) during follow-up

	**Group**^**a**^			***P*-value**
	**I (n = 33)**	**II (n = 30)**	**III (n = 29)**	
UICC stage III, n	10	8	8	
UICC stage IV, n	23	22	21	
UICC stage IVa, n	19	20	18	
UICC stage IVb, n	4	2	3	
Primary tumor n (%)^b^				
CR	16 (48.5)	5 (16.7)^d^	5 (17.2)^d^	0.006^b^
UICC stage III	6 (60.0)	2 (25.0)	1 (12.5)	0.098
UICC stage IV	10 (43.5)	3 (13.6)	4 (19.0)	0.051
PR	11 (33.3)	11 (36.7)	10 (34.5)	0.961
UICC stage III	2 (20.0)	3 (37.5)	4 (50.0)	0.450
UICC stage IV	9 (39.1)	8 (36.4)	6 (28.6)	0.572
SD or PD	6 (18.2%)	14 (46.7)^d^	14 (48.3)^d^	0.020^b^
UICC stage III	2 (20.0)	3 (37.5)	3 (37.5)	0.645
UICC stage IV	4 (17.4)	11 (50.0)^d^	11 (50.0)^d^	0.028^b^
Recurrence	3 (9.1)	6 (20.0)	7 (24.1)	0.267
Death	16 (48.5)	20 (66.7)	22 (75.9)	0.050
Side effects, n (%)^c^				
Flu-like symptoms	27 (81.8%)	23 (76.7%)	16 (55.2)	0.051
UICC stage III	9 (90.0%)	4 (50.0%)	4 (50.0%)	0.113
UICC stage IV	18 (78.3%)	19 (86.4%)	12 (57.1%)	0.078
Bone marrow suppression	12 (36.4%)	0 (0.0%)	11 (37.9%)	0.001^c^
UICC stage III	2 (20.0%)	0 (0.0%)	2 (25.0%)	0.506
UICC stage IV	10 (43.5%)	0 (0.0%)	9 (42.9%)	0.001^c^

Abbreviations: CR, complete response; PR, partial response; PD, progressive disease; SD, stable disease; UICC, International Union Against Cancer.

^a^Group I: chemotherapy and rAD-p53; Group II: rAD-p53; Group III: chemotherapy.

^b^Statistically significant difference (*P* < 0.05) between the three treatment groups.

^c^Statistically significant (*P* < 0.05). *P*-values are based on Fisher’s exact test for UICC stage III and on the χ^2^ test for UICC stage IV.

^d^Statistically significant difference between the indicated treatment group and group I.

**Figure 2:**
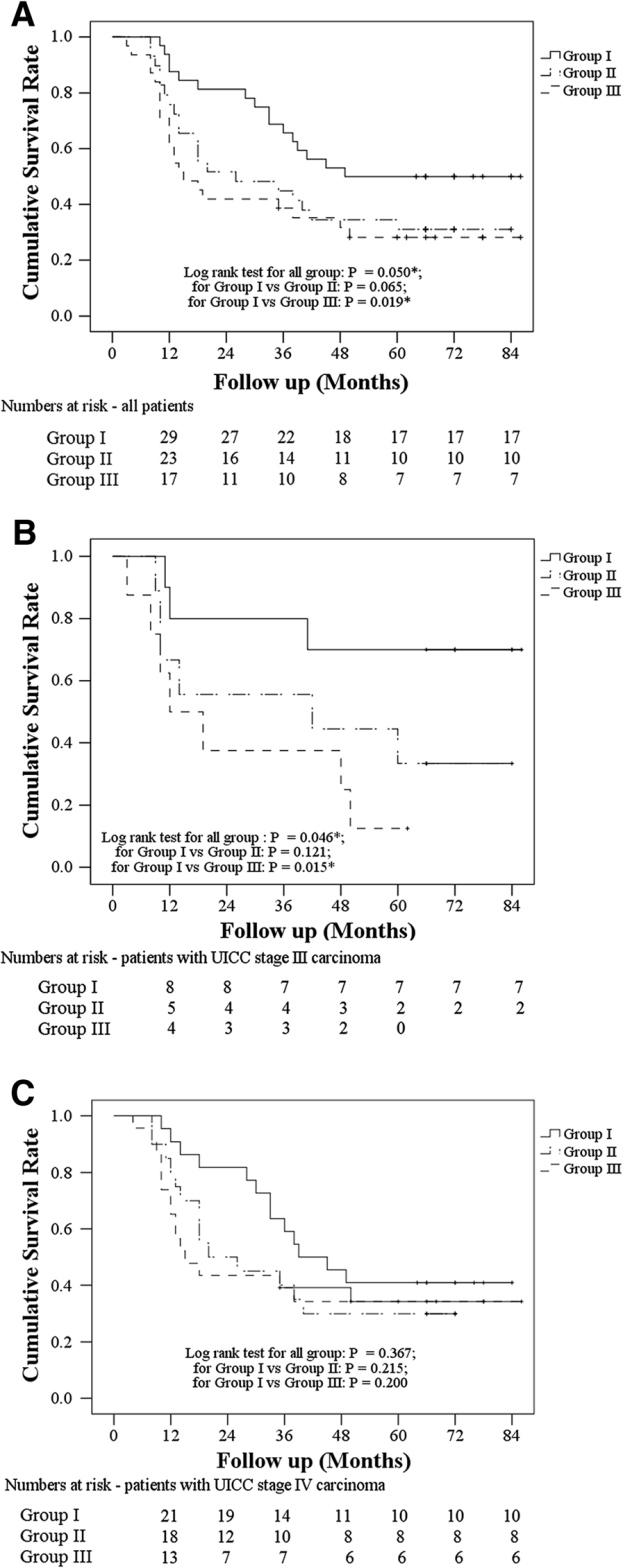
**Kaplan-Meier survival curves.** Kaplan-Meier survival curves of patients after treatment with chemotherapy and recombinant adenovirus (rAD)-p53 (group I), rAD-p53 (group II), or chemotherapy (group III) are shown **(A)** for all patients, **(B)** patients with International Union Against Cancer (UICC) stage III carcinoma, and **(C)** patients with UICC stage IV carcinoma. The numbers under the curve are the numbers of patients at risk in each group at each specific follow-up point.

The median length of follow-up was 36 months (range 3 to 86 months). During follow-up, 16 patients in group I, 20 in group II, and 22 in group III died. There were three patients in group I, six in group II, and then in group III with post-treatment recurrence. Survival was evaluated for the entire population, and then for the patients with stage III or stage IV disease. A log-rank test for survival rate comparing the three groups indicated that group I had a significantly higher survival rate than group III (*P* = 0.050 for all groups; *P* = 0.065 for group I versus group II; *P* = 0.019 for group I versus group III) (Figure 2A). The survival rate of patients with stage III oral cancer was significantly higher in group I than in group III (Figure 2B) (*P* = 0.046 for all groups; *P* = 0.121 for group I versus group II; *P* = 0.015 for group I versus group III). However, the survival rate of patients with stage IV did not significantly differ between the three groups (Figure 2C) (*P* = 0.367 for all groups; *P* = 0.215 for group I versus group II; *P* = 0.200 for group I versus group III).

Anti-rAd5 neutralizing antibody titers increased or became positive in all patients in groups I and II after the first infusion with rAd-p53 (group I: basal level 176 ± 103 to week 2 of treatment 2129 ± 1198; and group II: basal level 168 ± 101 to week 2 of treatment 2137 ± 1173, respectively). Antibody titers increased after administration of subsequent cycles of treatment; however, the anti-tumor activity of rAd-p53 was not affected by the neutralizing antibodies. No replication-deficient virus was detected in serum, urine, or sputum, suggesting that intra-arterial infusion did not result in systemic distribution.

**Figure 3:**
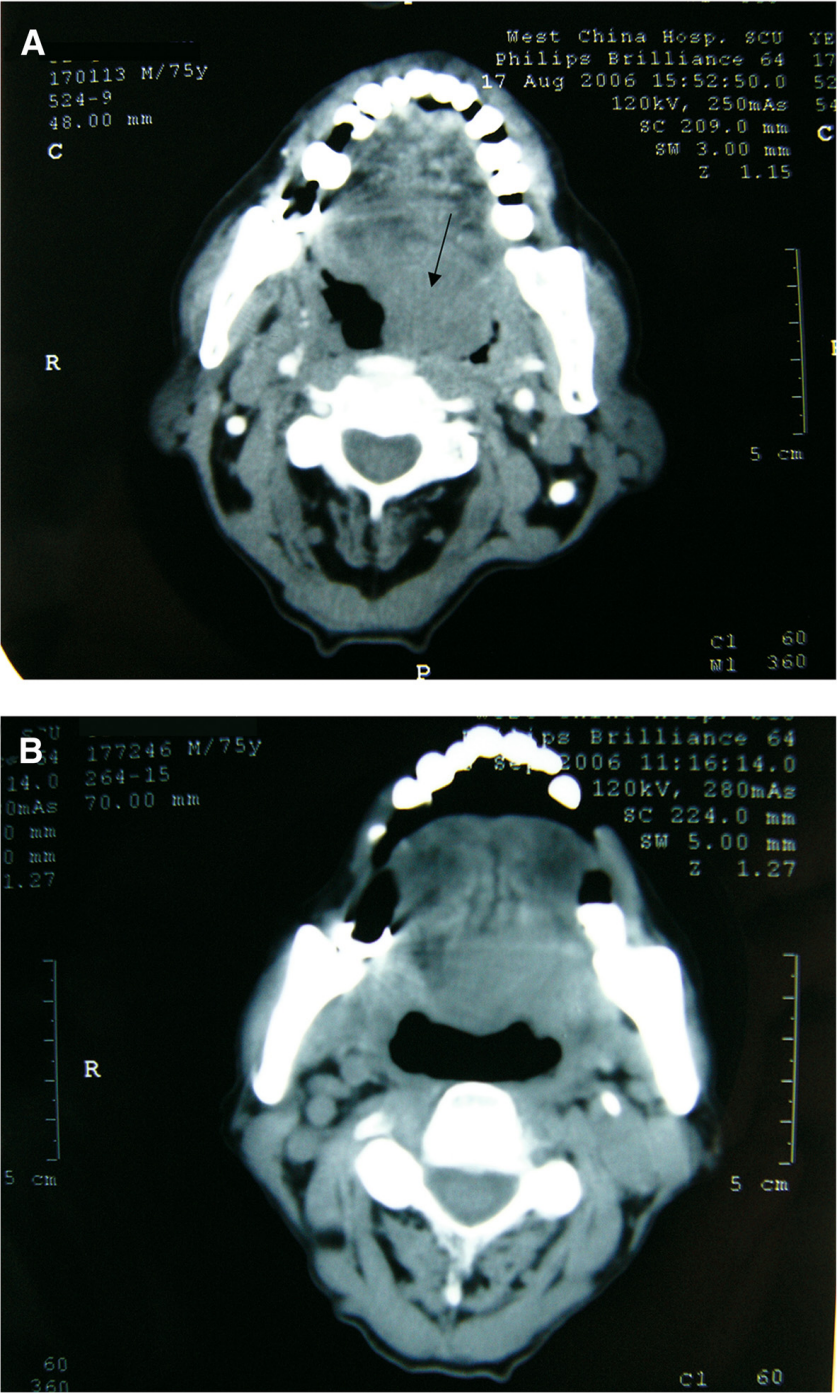
**Computed tomography (CT) images of a 75-year-old male patient from group I who had a complete response after the combined treatment (chemotherapy and recombinant adenovirus (rAd)-p53).** Radiographic CT images of **(A)** the pre-treatment lesion and **(B)** the same region after the treatment.

An example of a patient with CR from group I is presented in Figure 3. This 75-year-old male patient from group I had CR of the primary tumor mass after the treatment with chemotherapy and rAd-p53. Comparison of his pre-treatment and post-treatment CT images show that the pre-treatment tumor lesion (Figure 3A) had completely disappeared after the treatment (Figure 3B). In addition, the patient reported no discomfort.

### Adverse events

The most common treatment-related toxicities were transient flu-like symptoms (66 events) and bone marrow suppression (23 events). The majority of all groups experienced flu-like symptoms, especially the groups receiving rAd-p53 (Table 2). The incidences of flu-like symptoms were not significantly different between the three treatment groups (*P* = 0.051). In addition, gene therapy alone (group II) did not induce bone marrow suppression, suggesting that this side effect was due to the chemotherapy.

The mean change from baseline in WBC count, and levels of lymphocytes, neutrophil granulocytes, and MID was significantly different between the three groups for UICC stage III and IV (Table 3, *P* < 0.05). Patients receiving chemotherapy showed significant declines in whole blood cell counts due to bone marrow suppression (group I, -1.17 ± 0.65 10^2^/μl; group III, -2.13 ± 0.75 10^2^/μl). A change in WBC count was not found in the group II patients (Table 3). Interestingly, the WBC declines in group I (rAd-p53+ chemotherapy) were significantly less than in group III (chemotherapy) (Table 3).

**Table 3:** Change in whole blood cells, lymphocyte, neutrophil levels, MID and platelet counts between pre-treatment and the post-treatment for the three groups (n = 92)

	**Group**^**a**^			***P*-value**
	**I (n = 33)**	**II (n = 30)**	**III (n = 29)**	
UICC stage III, n	10	8	8	
UICC stage IV, n (%)	23 (70)	22 (73)	21 (72)	
Whole blood cell count, mean ± SD				
UICC stage III	-0.11 ± 0.54^e^	-0.04 ± 0.29^c^	-2.34 ± 0.74^cde^	<0.001^b^
UICC stage IV	-1.19 ± 0.73^e^	-0.03 ± 0.35^c^	-2.06 ± 0.75^cde^	<0.001^b^
Lymphocyte levels				
UICC stage III	-0.68 ± 0.34^e^	0.21 ± 0.59^c^	-0.91 ± 0.41^de^	<0.001^b^
UICC stage IV	-0.77 ± 0.46^e^	0.23 ± 0.54^c^	-1.02 ± 0.57^de^	<0.001^b^
Neutrophil granulocytes, mean ± SD				
UICC stage III	-0.38 ± 0.63	-0.33 ± 0.47	-1.16 ± 0.56^de^	0.015^b^
UICC stage IV	-0.35 ± 0.50^e^	-0.36 ± 0.52^e^	-0.86 ± 0.63^cde^	0.004^b^
MID, mean ± SD				
UICC stage III	-0.15 ± 0.22	0.10 ± 0.24	-0.29 ± 0.25^de^	0.013^b^
UICC stage IV	-0.07 ± 0.23	0.05 ± 0.23	-0.17 ± 0.18^de^	0.005^b^
Platelet counts, mean ± SD				
UICC stage III	-22.20 ± 70.85	-23.00 ± 22.01^e^	-25.57 ± 27.09^e^	0.990
UICC stage IV	-54.09 ± 35.46^e^	-7.68 ± 19.49^c^	-23.91 ± 29.30^cde^	<0.001^b^

Abbreviations: MID, minimum inhibitory dilution (which measures rare cells and precursor white cells); UICC, International Union Against Cancer.

^a^Group I: chemotherapy and rAd-p53; group II: rAd-p53; group III: chemotherapy.

^b^Statistically significant difference between the three treatment groups.

^c^Statistically significant difference between the indicated treatment group and group I.

^d^Statistically significant difference between groups II and III.

^e^Statistically significant difference between pre-treatment and post-treatment stages of each group by paired t-test.

### p53, Bax, and Bcl2 expression

**Figure 4:**
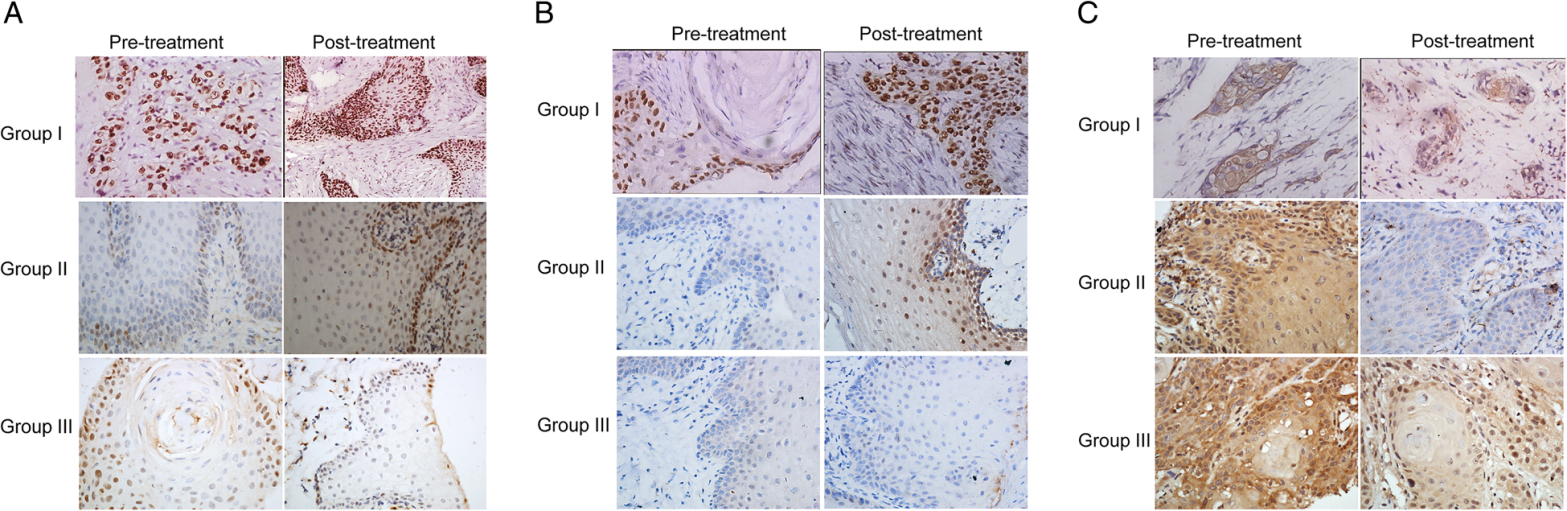
**Immunohistochemical staining of p53, Bax, and Bcl-2 in the primary tumor tissues.** Biopsies of primary lesion from patients in groups I, II and III were taken 3 days before the treatment (pre-treatment) and 3 days after the treatment (post-treatment), and processed for immunoreactivity to **(A)** p53, **(B)** Bax, and **(C)** Bcl-2. The p53 staining post-treatment is presented at ×100 magnification, whereas all other panels are presented at ×400 magnification.

Assessing the delivery of rAd-p53 to the tumor mass can be challenging because of the location of the biopsy. One option is to assess any changes in the expression of two transcriptional targets of p53, namely, Bax (a protein induced by p53) and Bcl-2 (a protein that is down-regulated by p53) [45]. We examined the change in p53, Bax, and Bcl-2 expression in the biopsy specimens of the primary oral carcinoma tumor tissue between pre-treatment and post-treatment stages by semi-quantitative immunostaining analysis (Figure 4). In group I, high expression of p53 was detected after treatment (Figure 4A). Most primary tumor masses before treatment showed Bcl-2 expression in at least 25% of cells, but Bax expression in less than 10% of tumor cells (Table 1). The distribution of Bax and Bcl-2 before treatment was similar for the three groups (Table 1). Higher Bax levels were found after treatment in the primary tumor mass of 28/33 patients in group I (84.8%) and 27/30 patients in group II (90.0%) (Figure 4B, Table 4), but only in 1/29 in group III. Higher Bax protein intensity was observed in all 19 patients achieving CR in groups I and II after receiving rAd-p53 treatment. Conversely, Bcl-2 immunostaining decreased in 30/33 patients (90.9%) in group I and 24/30 patients (80.0%) in group II (Figure 4C, Table 4). No enhancement of Bax immunoreactivity was observed in 8 of 20 patients in groups I and II who did not show CR response to rAd-p53 treatment.

**Table 4:** Change in immunostaining of carcinoma cells between pre-treatment and post-treatment, assessed by semi-quantitative analysis (n = 92)

	**Group**^**a**^			***P*-value**
	**I (n = 33)**	**II (n = 30)**	**III (n = 29)**	
Bax immunostaining, n (%)				
Decrease	0 (0.0)	0 (0.0)	1 (3.4)	<0.001^b^
No change	5 (15.2)	3 (10.0)	27 (93.1)	
Increase	28 (84.8)	27 (90.0)	1 (3.4)	
Bcl-2 immunostaining, n (%)				
Decrease	30 (90.9)	24 (80.0)	8 (27.6)	<0.001^b^
No change	3 (9.1)	4 (13.3)	18 (62.1)	
Increase	0 (0.0)	2 (6.7)	3 (10.3)	

^a^Group I: chemotherapy and rAD-*p53*; group II: rAD-*p53*; group III: chemotherapy.

^b^*P*-values are based on Fisher’s exact test.

## Discussion and conclusions

This single-center, randomized, placebo-controlled, double-blind, phase III clinical study showed that intra-arterial infusion of rAd-p53 and chemotherapy is associated with significantly better outcomes than either treatment alone, with a three-fold advantage in CR rate of the primary tumor mass. The survival rate was significantly higher in group I patients (intra-arterial rAd-p53 plus chemotherapy) with UICC III oral cancer. Patients selected for this study did not undergo prior surgery or radiotherapy, and treatment outcomes were recorded before any post-treatment local management by surgery. For complete disclosure, we state that after recording of all outcomes, the percentage of patients who underwent surgery was similar in group I (n = 10, 30.3%), group II (n = 7, 23.3%) and group III (n = 7, 24.1%) (*P* = 0.787), and two patients received both surgery and radiotherapy. Thus, survival rates and other outcomes were related to the study treatments, and were not influenced by other interventions. Immunohistochemical analysis of biopsy specimens showed increased levels of staining for wt-p53 protein in those patients who received infusion of rAd-p53. Exogenous p53 protein produced by rAd-p53 uptake, and expression in the primary lesion appeared to be functionally active in the approximately 80% of patients who had higher Bax and lower Bcl-2 expression in the post-treatment primary tumor samples. Our results emphasize that rAd-p53 activity is intimately related to outcome. These findings are similar to those of Li *et al*., who observed an increase in p21 in 14 of 22 rAd-p53-treated tumor samples, a decline in bcl-2 staining in 8 of 22 samples, and detectable apoptosis (by terminal dUTP nick end labeling (TUNEL) staining) in 18 of 22 samples [15], suggesting that rAd-p53 intra-tumoral administration and p53 expression increased tumor cell death by apoptosis in most samples (81.8%). As an increase in Bax protein level was observed in all our patients achieving CR in groups I and II, we infer that p53 was expressed after rAd-p53 administration, and led to tumor cell death in these patients. The higher Bax expression is consistent with the rAd-p53-induced apoptosis observed by Li *et al*. [15]. By contrast, Bax immunoreactivity remained unchanged in approximately half of the patients in groups I and II who did not show CR to rAd-p53 treatment. Thus, these data suggest that the increase in Bax expression in the primary lesion was associated with the functional activity of the exogenous wt-p53 protein.

The p53 protein is a key element in the apoptotic signaling cascade, and a mutation in the p53 gene reduces the susceptibility of a cell to apoptosis. Alterations in p53 can occur early in carcinogenesis, and are maintained during progression to overt malignancy. Flooding this mutant gene with wt p53 via adenovirally mediated p53 gene therapy has provided a modest therapeutic benefit. Sensitivity of head and neck SCC (HNSCC) to rAd-p53 therapy was associated with p53 status [12]. High expression of mutated p53 in patients with recurrent HNSCC significantly decreased efficacy and tumor response of Ad-p53 gene therapy [12]. Conversely, low levels of p53 protein were favorable for p53 gene therapy in patients with recurrent HNSCC [12]. As expected, the p53 profiles predictive of efficacy of p53 gene therapy did not predict methotrexate response [12]. In the current study, we detected wt-p53 in the patients (Figure 4A), but we did not examine the mutated p53 expression profile.

Compared with previous studies using intratumoral or intravenous infusion of rAd-p53 [10,11,36], this study achieved higher overall clinical response rates with intra-arterial administration. A comparison with our Kaplan-Meier plot shows that group II (rAd-p53 alone) was associated with longer median survival time than in the study of Clayman *et al*. [10,11]. Direct comparison of outcomes is constrained because of differences in study populations (for example, our study excluded patients with refractory disease). However, the following factors may have contributed to our positive results. First, we delivered rAd-p53 and/or chemotherapy through selective intra-arterial infusion in a retrograde manner, which, compared with intravenous infusion, more accurately targets the tumor, increases the local effective therapeutic dosage, may distribute the rAd-p53 more evenly throughout the tumor, and is associated with fewer side effects. Second, the blood vessels feeding oral tumors are typically more anatomically apparent than in healthy tissue, which facilitates accurate delivery of drug to the target area. Indocyanine green fluorescence improve identification of the blood supply to the tumor [46]. Third, because greater rAd-p53 doses yielded better responses [10], improved dose delivery via intra-arterial infusion may have contributed to the higher survival rate in patients with stage III oral carcinoma. Fourth, the physical status of our patients may have also contributed to survival, as our population was treatment-naive and did not have any morbidities resulting from toxicity or serious side effects associated with previous chemoradiotherapy or surgery.

The combination of intra-arterial rAd-p53 and chemotherapy showed a significantly greater treatment response and survival benefit compared with either treatment alone, consistent with a synergistic interaction. Combination of rAd-p53 with other forms of treatment has also significantly improved CR and patient survival. Weinrib *et al*. [47] suggested that rAd-p53 and cisplatin interact in an additive manner to kill C666-1 and CNE-1 cells. Somatic cell DNA damage inflicted by chemotherapy does not increase the risk of adenovirus DNA integration into genome. On the contrary, the non-specific induced DNA damage helps to activate the wt-p53 protein introduced by rAd-p53, with consequent synergistic effect [27]. If wt-p53 function is lost by down-regulation or mutation, chemotherapy is less effective [45], which may explain the increased effectiveness of combination therapy. In this study, we did not see CR for cervical (neck) lesions in any of the groups, which might be related to the small number of patients. Despite the lack of statistical significance, we observed that for neck metastases, 15/33 group I patients (45.5%) achieved PR and 9/33 (27.3%) SD whereas 10/30 group II patients (33.3%) achieved PR and 8/30 (26.7%) SD, and (10/29 group III patients (34.5%) had PR and 5/29 (17.2%) SD. The higher percentage of PR in group I compared with groups II or III did not reach statistical significance. We propose that the following three criteria are necessary to enhance the efficacy of the treatment: 1) regular and sufficient administration of rAd-p53; 2) adequate local concentration of rAd-p53; and 3) combination of gene therapy with chemotherapy.

Results from other trials of rAd-p53 therapy have shown that rAd-p53 treatment appears safe and well-tolerated [10,11,15,36,48]. The limited dataset and statistical power in our small pilot study did not detect any novel safety concerns. Approximately 82% of patients who received intra-arterial infusion of rAd-p53 experienced flu-like symptoms and/or bone marrow suppression. The adverse events such as flu-like symptoms appeared to be associated with the adenovirus delivery vehicle itself rather than with the encoded exogenous wt-p53 gene, similar to previous studies [48]. A noteworthy finding of our study was that bone marrow suppression was significantly less common with rAd-p53 plus chemotherapy compared with chemotherapy alone. We propose that the mechanisms by which wt-p53 protein may reduce the chemotherapy-induced toxicity involves at least one of the following three pathways: 1) interaction of p53 protein with DNA helicase; 2) up-regulation of ribonucleotide reductase (p53R2) by p53; or 3) the 3′→ 5′ exonuclease activity of the p53 protein. However, further studies are warranted.

This study had several limitations. Although larger than previous investigations [10,11], this small clinical study (fewer than 40 patients were randomized to each group) of gene therapy for SCC was conducted at a single center. The low incidence of advanced (stage III/IV) oral carcinoma precluded recruitment of a larger study population. Nevertheless, the findings and protocol used in this study can be used to aid in designing a future larger multi-center clinical trial. Second, our study design did not contain a control group that received dosing through intratumoral or intravenous administration for direct comparison. Third, because the chemotherapeutic agent is a prodrug that must be metabolized in the liver into the active drug, intra-arterial delivery may also have reduced the effectiveness of chemotherapy. Fourth, the patients had a range of tumor characteristics (location, classification, stage). However, these potential sources of heterogeneity were minimized because the distribution of SCC was similar in the three groups. Disease heterogeneity did not appear to account for differences in outcomes among the three groups. Fifth, only patients with advanced disease (stage III or IV) who refused or were not suitable for surgery were enrolled. No patients with stage I or II disease were included, as most would have received surgical treatment. A treatment-naive population with advanced SCC does not reflect current, routine presentation in advanced industrialized countries. However, our study does provide proof of principle that intra-arterial infusion of rAd-p53 with chemotherapy has clinical benefit in advanced oral cancer. We are currently performing a clinical trial in which patients with advanced oral carcinoma receive chemotherapy and gene therapy after surgery.

In summary, the combination of gene therapy (rAD-p53) and chemotherapy produced significantly higher CR rates than either gene therapy alone or chemotherapy alone. These findings are consistent with a synergistic interaction, which has also been described in publications with other types of tumors. Interestingly, no significant adverse effects were found, and bone marrow suppression was significantly less common in group I (gene therapy plus chemotherapy) than in group III (chemotherapy alone). This study also demonstrated that the p53 protein delivered by gene therapy is functionally active in 80% of cases (based on analysis of Bax and Bcl-2 expression in primary tumor). This pilot study provides the proof of benefits of intra-arterial infusion for cancer gene therapy. These results are noteworthy, and suggest that intra-arterial infusion of combined rAd-p53 and chemotherapy is a viable strategy for the management of oral SCC, and may represent an alternative to chemoradiotherapy and surgery, which is the current standard of care.

## Competing interest

None of the authors have any conflict of interest with the manufacturer of recombinant adenoviral p53 (Gendicine^®^).

## Authors’ contributions

YL was responsible for study concepts, definition of intellectual content, clinical, and experimental studies, and manuscript preparation. L-JL is the guarantor of the integrity of the entire study, and was responsible for study design, manuscript editing, and review. L-JW was responsible for clinical studies, data analysis, and statistical analysis. ZZ was responsible for clinical studies, manuscript editing, and review. NG was responsible for clinical studies, manuscript editing, and review. C-YL was responsible for literature research, experimental studies, and data analysis. Y-DH was responsible for experimental studies, data analysis, and manuscript preparation. BH was responsible for clinical studies and statistical analysis. All authors read and approved the final manuscript.

## Pre-publication history

The pre-publication history for this paper can be accessed here:

http://www.biomedcentral.com/1741-7015/12/16/prepub

## References

[B1] Bagnardi V Blangiardo M La Vecchia C Corrao G Alcohol consumption and the risk of cancer: a meta-analysis Alcohol Res Health 2001 25 263 270 11910703 PMC6705703

[B2] Lewin F Norell SE Johansson H Gustavsson P Wennerberg J Biorklund A Rutqvist LE Smoking tobacco, oral snuff, and alcohol in the etiology of squamous cell carcinoma of the head and neck: a population-based case-referent study in Sweden Cancer 1998 82 1367 1375 10.1002/(SICI)1097-0142(19980401)82:7<1367::AID-CNCR21>3.0.CO;2-3 9529030

[B3] Fakhry C Gillison ML Clinical implications of human papillomavirus in head and neck cancers J Clin Oncol 2006 24 2606 2611 10.1200/JCO.2006.06.1291 16763272 PMC4696042

[B4] National Comprehensive Cancer Network (NCCN), Clinical Practice Guidelines in Oncology Head and Neck Cancers In Book Clinical Practice Guidelines in Oncology 2010 Head and Neck Cancers. V.1 Available from http://www.nccn.org. Accessed April 20, 2010

[B5] Brown AE Langdon JD Management of oral cancer Ann R Coll Surg Engl 1995 77 404 408 8540656 PMC2502477

[B6] Hyde NC Prvulovich E Newman L Waddington WA Visvikis D Ell P A new approach to pre-treatment assessment of the N0 neck in oral squamous cell carcinoma: the role of sentinel node biopsy and positron emission tomography Oral Oncol 2003 39 350 360 10.1016/S1368-8375(02)00121-5 12676254

[B7] Forastiere A Koch W Trotti A Sidransky D Head and neck cancer N Engl J Med 2001 345 1890 1900 10.1056/NEJMra001375 11756581

[B8] Lippman SM Hong WK Second malignant tumors in head and neck squamous cell carcinoma: the overshadowing threat for patients with early-stage disease Int J Radiat Oncol Biol Phys 1989 17 691 694 10.1016/0360-3016(89)90126-0 2674081

[B9] Edelman J Nemunaitis J Adenoviral p53 gene therapy in squamous cell cancer of the head and neck region Curr Opin Mol Ther 2003 5 611 617 14755887

[B10] Clayman GL El-Naggar AK Lippman SM Henderson YC Frederick M Merritt JA Zumstein LA Timmons TM Liu TJ Ginsberg L Roth JA Hong WK Bruso P Goepfert H Adenovirus-mediated p53 gene transfer in patients with advanced recurrent head and neck squamous cell carcinoma J Clin Oncol 1998 16 2221 2232 9626224 10.1200/JCO.1998.16.6.2221

[B11] Clayman GL Frank DK Bruso PA Goepfert H Adenovirus-mediated wild-type p53 gene transfer as a surgical adjuvant in advanced head and neck cancers Clin cancer Res 1999 5 1715 1722 10430074

[B12] Nemunaitis J Clayman G Agarwala SS Hrushesky W Wells JR Moore C Hamm J Yoo G Baselga J Murphy BA Menander KA Licato LL Chada S Gibbons RD Olivier M Hainaut P Roth JA Sobol RE Goodwin WJ Biomarkers predict p53 gene therapy efficacy in recurrent squamous cell carcinoma of the head and neck Clin Cancer Res 2009 15 7719 7725 10.1158/1078-0432.CCR-09-1044 19996201

[B13] Ries JC Schreiner D Steininger H Girod SC p53 mutation and detection of p53 protein expression in oral leukoplakia and oral squamous cell carcinoma Anticancer Res 1998 18 2031 2036 9677462

[B14] Sakai E Tsuchida N Most human squamous cell carcinomas in the oral cavity contain mutated p53 tumor-suppressor genes Oncogene 1992 7 927 933 1570156

[B15] Li Y Li LJ Zhang ST Wang LJ Zhang Z Gao N Zhang YY Chen QM In vitro and clinical studies of gene therapy with recombinant human adenovirus-p53 injection for oral leukoplakia Clin Cancer Res 2009 15 6724 6731 10.1158/1078-0432.CCR-09-1296 19861457

[B16] Johnstone RW Ruefli AA Lowe SW Apoptosis: a link between cancer genetics and chemotherapy Cell 2002 108 153 164 10.1016/S0092-8674(02)00625-6 11832206

[B17] Liloglou T Scholes AG Spandidos DA Vaughan ED Jones AS Field JK p53 mutations in squamous cell carcinoma of the head and neck predominate in a subgroup of former and present smokers with a low frequency of genetic instability Cancer Res 1997 57 4070 4074 9307295

[B18] Gao WM Mady HH Yu GY Siegfried JM Luketich JD Melhem MF Keohavong P Comparison of p53 mutations between adenocarcinoma and squamous cell carcinoma of the lung: unique spectra involving G to A transitions and G to T transversions in both histologic types Lung Cancer 2003 40 141 150 10.1016/S0169-5002(03)00035-7 12711114

[B19] Tetu B Brisson J Plante V Bernard P p53 and c-erbB-2 as markers of resistance to adjuvant chemotherapy in breast cancer Modern Pathol 1998 11 823 830 9758361

[B20] Brand K Klocke R Possling A Paul D Strauss M Induction of apoptosis and G2/M arrest by infection with replication-deficient adenovirus at high multiplicity of infection Gene Ther 1999 6 1054 1063 10.1038/sj.gt.3300914 10455408

[B21] Spitz FR Nguyen D Skibber JM Meyn RE Cristiano RJ Roth JA Adenoviral-mediated wild-type p53 gene expression sensitizes colorectal cancer cells to ionizing radiation Clin Cancer Res 1996 2 1665 1671 9816114

[B22] Swisher SG Roth JA Nemunaitis J Lawrence DD Kemp BL Carrasco CH Connors DG El-Naggar AK Fossella F Glisson BS Adenovirus-mediated p53 gene transfer in advanced non-small-cell lung cancer J Natl Cancer Inst 1999 91 763 771 10.1093/jnci/91.9.763 10328106

[B23] Parker LP Wolf JK Price JE Adenoviral-mediated gene therapy with Ad5CMVp53 and Ad5CMVp21 in combination with standard therapies in human breast cancer cell lines Ann Clin Lab Sci 2000 30 395 405 11045764

[B24] Modesitt SC Ramirez P Zu Z Bodurka-Bevers D Gershenson D Wolf JK In vitro and in vivo adenovirus-mediated p53 and p16 tumor suppressor therapy in ovarian cancer Clin Cancer Res 2001 7 1765 1772 11410518

[B25] Lang FF Bruner JM Fuller GN Aldape K Prados MD Chang S Berger MS McDermott MW Kunwar SM Junck LR Phase I trial of adenovirus-mediated p53 gene therapy for recurrent glioma: biological and clinical results J Clin Oncol 2003 21 2508 2518 10.1200/JCO.2003.11.138 12839017

[B26] Schuler M Herrmann R De Greve JL Stewart AK Gatzemeier U Stewart DJ Laufman L Gralla R Kuball J Buhl R Adenovirus-mediated wild-type p53 gene transfer in patients receiving chemotherapy for advanced non-small-cell lung cancer: results of a multicenter phase II study J Clin Oncol 2001 19 1750 1758 11251006 10.1200/JCO.2001.19.6.1750

[B27] Buller RE Runnebaum IB Karlan BY Horowitz JA Shahin M Buekers T Petrauskas S Kreienberg R Slamon D Pegram M A phase I/II trial of rAd/p53 (Sch 58500) gene replacement in recurrent ovarian cancer Cancer Gene Ther 2002 9 553 566 10.1038/sj.cgt.7700472 12082455

[B28] Moon C Oh Y Roth JA Current status of gene therapy for lung cancer and head and neck cancer Clin Cancer Res 2003 9 5055 5067 14613982

[B29] Weill D Mack M Roth J Swisher S Proksch S Merritt J Nemunaitis J Adenoviral-mediated p53 gene transfer to non-small cell lung cancer through endobronchial injection Chest 2000 118 966 970 10.1378/chest.118.4.966 11035664

[B30] Pagliaro LC Keyhani A Williams D Woods D Liu B Perrotte P Slaton JW Merritt JA Grossman HB Dinney CP Repeated intravesical instillations of an adenoviral vector in patients with locally advanced bladder cancer: a phase I study of p53 gene therapy J Clin Oncol 2003 21 2247 2253 10.1200/JCO.2003.09.138 12805322

[B31] Han DM Huang ZG Zhang W Yu ZK Wang Q Ni X Chen XH Pan JH Wang H Effectiveness of recombinant adenovirus p53 injection on laryngeal cancer: phase I clinical trial and follow up Zhonghua Yi Xue Za Zhi 2003 83 2029 2032 14703409

[B32] Chen CB Pan JJ Xu LY Recombinant adenovirus p53 agent injection combined with radiotherapy in treatment of nasopharyngeal carcinoma: a phase II clinical trial Zhonghua Yi Xue Za Zhi 2003 83 2033 2035 14703410

[B33] Zhang SW Xiao SW Liu CQ Sun Y Su X Li DM Xu G Cai Y Zhu GY Xu B Lu YY Treatment of head and neck squamous cell carcinoma by recombinant adenovirus-p53 combined with radiotherapy: a phase II clinical trial of 42 cases Zhonghua Yi Xue Za Zhi 2003 83 2023 2028 14703408

[B34] Pan JJ Zhang SW Chen CB Xiao SW Sun Y Liu CQ Su X Li DM Xu G Xu B Lu YY Effect of recombinant adenovirus-p53 combined with radiotherapy on long-term prognosis of advanced nasopharyngeal carcinoma J Clin Oncol 2009 27 799 804 10.1200/JCO.2008.18.9670 19103729

[B35] Alemany R Cancer selective adenoviruses Mol Aspects Med 2007 28 42 58 10.1016/j.mam.2006.12.002 17300834

[B36] Reid T Warren R Kirn D Intravascular adenoviral agents in cancer patients: lessons from clinical trials Cancer Gene Ther 2002 9 979 986 10.1038/sj.cgt.7700539 12522437 PMC7091735

[B37] Robbins KT Homma A Intra-arterial chemotherapy for head and neck cancer: experiences from three continents Surg Oncol Clin N Am 2008 17 919 933 xi 10.1016/j.soc.2008.04.015 18722926

[B38] Fuwa N Kodaira T Furutani K Tachibana H Nakamura T A new method of selective intra-arterial infusion therapy via the superficial temporal artery for head and neck cancer Oral Surg Oral Med Oral Pathol Oral Radiol Endod 2008 105 783 789 10.1016/j.tripleo.2007.07.031 18206406

[B39] Wu CF Huang CJ Chang KP Chen CM Continuous intra-arterial infusion chemotherapy as a palliative treatment for oral squamous cell carcinoma in octogenarian or older patients Oral Oncol 2010 46 559 563 10.1016/j.oraloncology.2010.04.006 20538502

[B40] Fuwa N Kodaira T Furutani K Tachibana H Nakamura T Nakahara R Tomoda T Inokuchi H Daimon T Intra-arterial chemoradiotherapy for locally advanced oral cavity cancer: analysis of therapeutic results in 134 cases Br J Cancer 2008 98 1039 1045 10.1038/sj.bjc.6604272 18283309 PMC2275486

[B41] Kovacs AF Intra-arterial induction high-dose chemotherapy with cisplatin for oral and oropharyngeal cancer: long-term results Br J Cancer 2004 90 1323 1328 10.1038/sj.bjc.6601674 15054449 PMC2409693

[B42] Peng Z Current status of gendicine in China: recombinant human Ad-p53 agent for treatment of cancers Hum Gene Ther 2005 16 1016 1027 10.1089/hum.2005.16.1016 16149900

[B43] Zhang SW Xiao SW Liu CQ Sun Y Su X Li DM Xu G Zhu GY Xu B Recombinant adenovirus-p53 gene therapy combined with radiotherapy for head and neck squamous-cell carcinoma Zhonghua Zhong Liu Za Zhi 2005 27 426 428 16188130

[B44] Peng Z Points to Consider for Human Gene Therapy and Product Quality Control State Food and Drug Administration of China http://www.biopharminternational.com/biopharm/article/articleDetail.jsp?id=95486

[B45] Gurnani M Lipari P Dell J Shi B Nielsen LL Adenovirus-mediated p53 gene therapy has greater efficacy when combined with chemotherapy against human head and neck, ovarian, prostate, and breast cancer Cancer Chemother Pharmacol 1999 44 143 151 10.1007/s002800050959 10412949

[B46] Ohba S Yokoyama J Fujimaki M Ito S Kojima M Shimoji K Ikeda K Significant improvement in superselective intra-arterial chemotherapy for oral cancer by using indocyanine green fluorescence Oral Oncol 2012 48 1101 1105 10.1016/j.oraloncology.2012.08.007 22974717

[B47] Weinrib L Li JH Donovan J Huang D Liu FF Cisplatin chemotherapy plus adenoviral p53 gene therapy in EBV-positive and -negative nasopharyngeal carcinoma Cancer Gene Ther 2001 8 352 360 10.1038/sj.cgt.7700319 11477455

[B48] Zhang S Li Y Li L Zhang Y Gao N Zhang Z Zhao H Phase I study of repeated intraepithelial delivery of adenoviral p53 in patients with dysplastic oral leukoplakia J Oral Maxillofac Surg 2009 67 1074 1082 10.1016/j.joms.2008.06.079 19375021

